# The Impact of Temperature on the Leaves of *Ceratonia siliqua* L.: Anatomical Aspect, Secondary Metabolite Analysis, and Antimicrobial Activity of the Extracts

**DOI:** 10.3390/plants14040557

**Published:** 2025-02-11

**Authors:** Aikaterina L. Stefi, Evangelos Kalampokis, Georgia C. Ntroumpogianni, Iliana Katsiadrami, Theodora Nikou, Efstathios A. Katsifas, Dimitrios Gkikas, Nikolaos S. Christodoulakis, Maria Halabalaki

**Affiliations:** 1 Section of Botany, Department of Biology, Faculty of Sciences, National and Kapodistrian University of Athens, 15784 Athens, Greece; georgiantr@biol.uoa.gr (G.C.N.); ili_ka@hotmail.com (I.K.); skatsi@biol.uoa.gr (E.A.K.); dimgkikas@biol.uoa.gr (D.G.); nchristo@biol.uoa.gr (N.S.C.); 2 Division of Pharmacognosy and Natural Products Chemistry, Department of Pharmacy, National and Kapodistrian University of Athens, 15784 Athens, Greece; kalampokis-v@pharm.uoa.gr (E.K.); th-nikou@pharm.uoa.gr (T.N.); mariahal@pharm.uoa.gr (M.H.)

**Keywords:** carob tree, leaf extracts, environmental stress, ecophysiology, LC-HRMS/MS analyses, bioassays

## Abstract

*Ceratonia siliqua* L. (Fabaceae) is an evergreen sclerophyllous species that successfully overcomes the challenges of the Mediterranean climate. Commonly, biosynthesis of secondary metabolites is a major reaction of the plants thriving in the Mediterranean formations against temperature stress. Due to concerns about the climate crisis, we studied the impact of 6-day low (5 °C) and high (40 °C) temperature stress on young carob seedlings. In stressed plants, mainly the heat-treated, the leaves appear xeromorphic. Parameters of the physiology of the plants such as chlorophyll-a and -b, total phenolic content, and oxidative stress were measured and presented via Principal Component Analysis. Chlorophyll-a and -b contents are inferior in cold-stressed leaves while heat-stressed leaves accumulate more phenolics and experience higher oxidative stress as compared to their cold-stressed counterparts. The phytochemical profile of different extracts obtained from stressed carob leaves was identified so as to gain insight into metabolites produced under stress. Moreover, LC-HRMS/MS metabolomic workflow was utilized for the discovery of biomarkers, over- or under-regulated in stressed conditions. The antimicrobial activity of carob leaf extract fractions was assessed against six human pathogen strains and three phytopathogen bacterial strains. MeOH-H_2_O and dichloromethane (DCM) extracts presented notable activity against *Candida albicans* and *Saccharomyces cerevisiae*, while DCM extracts inhibited the growth of *Erwinia amylovora*. We may conclude that carob tree exposure to temperature stress does not have a significant influence on secondary metabolic pathways.

## 1. Introduction

Volumes have been written on the peculiarities of the Mediterranean climate, the evolution, and the adaptation of Mediterranean plants, both the evergreen sclerophylls and the seasonally dimorphic plants. The Mediterranean environment imposes on both plant communities two severe, qualitatively different, and timely separated stresses: the combined stress of drought, high temperature, and high light intensity during the summer months and freezing temperatures during the winter [1]. Concerning low temperatures, the Mediterranean climate does have a winter, which can readily be defined as any month with an average temperature below 15 °C. However, during the winter, although mild in general, the hours per year that the temperature falls below freezing (0 °C) should not exceed 3% of the total. This phenomenon, being a good index of the severity of winters, directly affects plant life [2].

It has been demonstrated that the distribution of the Mediterranean evergreen sclerophyllous formations is controlled by the winter cold in northern latitudes and the summer drought in southern habitats [3]. Therefore, plants thriving either in the chaparral (evergreen sclerophyllous) or in the phryganic (seasonally dimorphics) formations, have developed interesting adaptive strategies [4] to either evade or escape the above-mentioned environmental stresses.

**Figure 1:**
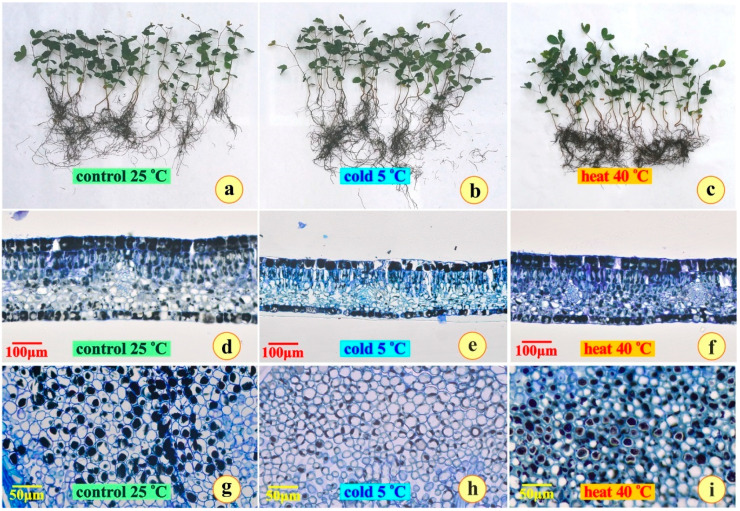
The plants of *Ceratonia siliqua* after the experiment; (**a**–**c**) Photographs of the whole plant for the control (**a**), cold-stressed (**b**), and heat-stressed (**c**) treatments after drying. Cross (**d**–**f**) and paradermal (**g**–**i**) sections of plastic-embedded leaf, stained with toluidine blue “O” for the control (**d**,**g**) cold-stressed (**e**,**h**) and heat-stressed (**f**,**i**) plants.

The carob or locust bean tree (*Ceratonia siliqua* L., Family: Fabaceae) (Figure 1a) is an emblematic species thriving among the evergreen sclerophyll formations [5]. It has developed remarkable adaptations to successfully overcome the overwhelmingly high temperatures and drought occurring during the summer months [4,6]. However, the natural distribution of *C. siliqua* in Greece, compared to other evergreen sclerophyllous species, hardly reaches the latitude of 38°, which is similar to the latitude of the city of Athens, while *Quercus coccifera* is distributed as far as the northern territories of Greece (latitude 39°76′) reaching up to 900 m [7]. The same is true for the “flagship” of the Mediterranean formations, the olive tree, which reaches the latitude of 39°64′ and an altitude of 500 m [7,8,9]. These evergreen sclerophylls are present in areas with higher altitudes while they are absent in areas with lower summer drought. This phenomenon is called the “Mediterranean shrub paradox”. This paradox was explained when the distribution data were compared to the cold gradient [3,7] while it was suggested that the limitations in the distribution of *C. siliqua* are mainly due to the susceptibility of this species to low temperatures [7].

In the hostile Mediterranean climate, the periods of favorable conditions, when plant growth can be promoted, are unpredictable and limited [4]. Furthermore, climatic change, one of the main challenges of this era, is accompanied by extreme weather conditions such as high temperatures, prolonged drought periods, severe storms and rainfalls, floods, snowless winters, and cold snap periods that pose immediate pressures on plants [10]. This has an impact not only on plants; the successive impact on crops creates an added new socioeconomic threat. Eventually, Mediterranean plants, although considered to be potentially very productive [11,12,13], fall short in yield [1,5]. This is due to the stress conditions of not only the long, arid Mediterranean summer but also the rapid “sweep” of the severe chilling conditions during winter. *C. siliqua*, as the distribution data along the Mediterranean basin indicate, seems to be well adapted to the summer stress conditions yet proves rather vulnerable to the winter cold stress (Table 1). There is a strong indication that the young tissues, leaves, and leaf buds exhibit reduced frost resistance [8,9] while the leaf primordia lack any protection scales [14].

Plants, in general, respond to a certain environmental stress in various ways. A prominent reaction among Mediterranean plants is the biosynthesis of secondary metabolites. Among them, a major group is that of the phenolic compounds [15,16,17] produced through the activation of the shikimic acid pathway [18]. Phenylalanine is one of the products of this pathway that, with the addition of malonyl-CoA, results in flavonoid production [19]. The accumulation of phenolics, even as metabolically terminal products, has been considered a very common feature of Mediterranean plants sustaining environmental stress [14,15,20,21,22,23,24,25,26]. Moreover, the effect of the stress conditions is also expressed by the decrease of photosynthetic pigment content because of the chloroplast breakdown caused by active oxygen species [27]. A balance can be reached by numerous secondary metabolites produced through the shikimic acid pathway, which have an antioxidant effect and increase the plant’s tolerance to the stress conditions [28,29].

Therefore, (a) willing to exploit the numerous pieces of data on the stress response of a Mediterranean evergreen sclerophyllous shrub; (b) considering that excessively low or high temperatures, which are new conditions in the Mediterranean regions provoked by climatic change, actually impose a severe stress to the plantlets of *C. siliqua*; (c) wondering if the winter cold stress has a similar effect as the summer stress; (d) regarding the shikimic acid pathway as the crucial mechanism for the relief of the oxidative potential of the cells and the origin of some interesting secondary metabolites; and (e) considering the carob tree as a plant of wide distribution and major commercial importance, we undertook this investigation in order to reveal the response of *C. siliqua* young plants after a quick exposure to considerably low and high temperatures and compare their reaction to that of plants used as “controls”. Thus, our primary aim is to detect any reactions concerning the development, morphology, anatomy, and biochemistry of the plant after a short period of stress (heat or cold). Additionally, to delve deeper into the molecular level, an extraction and analysis of the different specimens were performed. The antimicrobial activity against selected plant and human pathogens of the derived extracts was investigated and the most potent were characterized using different analytical platforms.

## 2. Results

The plants of the three groups (control, cold, and heat) were placed on filter paper and left to dry in an oven at 60 °C for three days. Some samples were randomly selected from the three treatments, and the total dry mass (shoot and root) was measured. The three groups appear to have developed similarly during the 6-day temperature stress period (Figure 1a–c). Concerning the anatomy of the leaves, after the observation of numerous sections of the three leaf types, it seems that the leaves of the plants grown as “controls” demonstrate the structure of the tissues common to most Mediterranean evergreen sclerophylls (Figure 1d). The epidermal cells are rich in phenolics. The palisade tissue appears compact both in the cross (Figure 1d) and paradermal (Figure 1g) sections. These leaves are thicker than the leaves of the other two groups. The leaves from the plants grown in the 5 °C chamber appear less xeromorphic (Figure 1e). Their thin epidermal cells, both adaxial and abaxial, and the moderately developed palisade tissue resulted in a thinner leaf (Figure 1e) accumulating less phenolics as well (Figure 1h). The leaves from the plants grown in the 40 °C chamber (Figure 1f) appear more xeromorphic. The epidermal cells are impregnated with densely stained phenolics while the palisade parenchyma, in longitudinal (Figure 1f) and cross sections (Figure 1i) appear very compact. The palisade cells accumulate phenolics (Figure 1h). The cross-sectioned leaves appear thinner than the “control” ones (compare Figure 1d to Figure 1f). Data on the thickness of the leaf tissues are given in Table 2. In particular, the thickness of the adaxial epidermis was statistically different between the cold- and heat-treated samples and the same is true for the total leaf thickness. The adaxial epidermis of the control plants is thicker than the cold-treated ones, while there is no statistically significant difference between the control and heat-treated plants. The total leaf thickness is greater in the control group than in the cold- and heat-treated groups. No statistically significant differences are noticed in the thickness of the abaxial epidermis and the thickness of the palisade parenchyma between the three groups.

**Figure 2:**
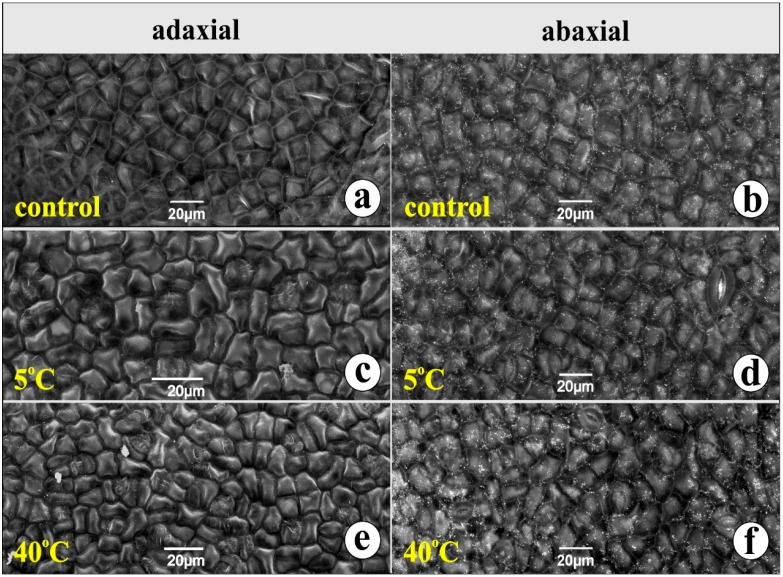
Scanning electron micrographs of the upper (adaxial) and lower (abaxial) epidermis from the leaves of *Ceratonia siliqua* after the end of the experiment; (**a**,**b**) the control plants; (**c**,**d**) the cold-stressed plants; and (**e**,**f**) the heat-stressed plants.

The upper (adaxial) and the lower (abaxial) epidermis of the leaf were thoroughly observed using scanning electron microscopy. Stomata do not exist on the upper epidermis (Figure 2a–c). The lower epidermis of all three leaf types exhibits many small anomocytic stomata (Figure 2b,d,f). Stomatal frequency was 234 ± 22 for the control plants, 242 ± 18 for the heat-treated plants, and 225 ± 12 for the cold-treated plants, with no statistical difference (*p* > 0.05).

**Figure 3:**
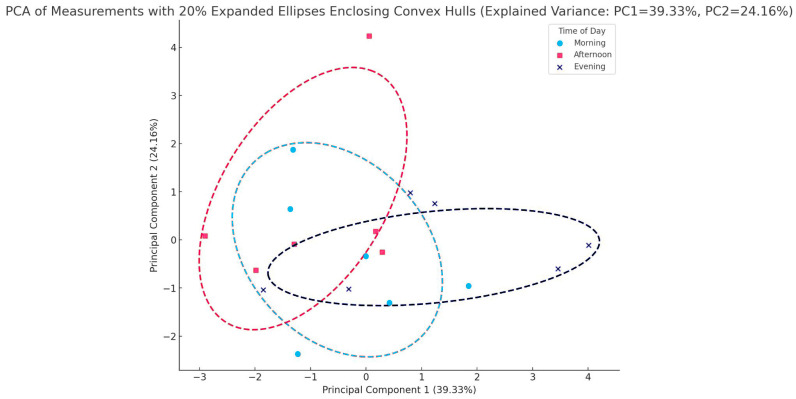
PCA analysis of MDA, TPC, Chl-a, and Chl-b in three clusters: morning samples, afternoon samples, and evening samples.

Chlorophyll concentration (Chl-a and Chl-b) was measured for 48 h after the end of the experiment. The malondialdehyde (MDA) content, indicating the oxidative stress the plants experience, was also defined, as well as the concentration of the total phenolic content (TPC). The samples for these parameters were collected at 08:00, 14:00, and 20:00 for day 1 and at 08:00, 14:00, and 20:00 for the day 2; the first sample was collected immediately after the end of the experiment (see Section 4). These parameters were analyzed using Principal Component Analysis (PCA) to detect any grouping of the specimens. The first PCA was utilized to explore temporal patterns in the dataset; the samples were grouped according to morning, afternoon, and evening timeframes, allowing for a nuanced examination of how measurements shift over the course of the day. The first two principal components, which together account for a substantial proportion of the total variance, provided a clear visual framework for examining these temporal patterns and revealed distinct separations among these time-based clusters (Figure 3). By plotting the data in the space of the first two principal components, the clusters corresponding to different times of day emerged naturally, with morning, afternoon, and evening samples displaying distinct spatial separations. To better represent the inherent variability within each temporal cluster, ellipses were drawn around the central distributions of the data points. These ellipses serve to highlight the range and density of measurements, capturing the intrinsic dispersion within each cluster.

Notably, the morning cluster exhibited a more concentrated distribution along the first principal component, suggesting that measurements taken during this period were relatively uniform. In contrast, the afternoon and evening clusters spread more broadly across the principal component space, indicating greater variability in measurements later in the day. This pattern may reflect diurnal rhythms, shifts in environmental conditions, or other time-dependent biological and ecological factors that influence the measurements.

**Figure 4:**
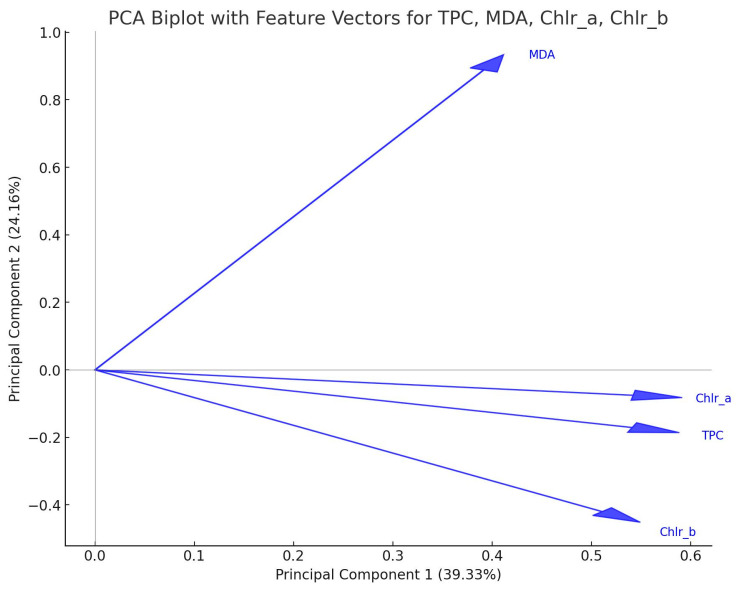
PCA biplot using data from TPC, MDA, Chl-a, and Chl-b measurements.

The second PCA analysis, a biplot, visualized the contributions of four features of stress physiology—TPC, MDA, Chl-a, and Chl-b—to the first two principal components (PC1 and PC2) derived from the dataset (Figure 4). Together, PC1 and PC2 account for a substantial share of the total variability (39.33% and 24.16%, respectively), offering a clear view of the dominant patterns within the data. In the biplot, each feature is represented by an arrow (or vector) radiating from the origin, with both the direction and length of the arrow providing crucial information about the feature’s influence on the principal components. When a feature vector is closely aligned with one of the principal component axes, it indicates that the feature strongly contributes to the variance explained by that component. For example, TPC and MDA load heavily on PC1, suggesting that their variations are central to the primary dimension of variability in the data. Meanwhile, Chl-a and Chl-b exhibit contributions to both PC1 and PC2, implying that their variability plays a role in multiple axes of differentiation within the dataset. The spatial arrangement and relative positioning of these vectors not only shed light on how strongly each feature influences the principal components but also suggest correlations among the features themselves. Vectors pointing in similar directions, for instance, are often associated with positive correlations, whereas those pointing in opposite directions may indicate inverse relationships.

**Figure 5:**
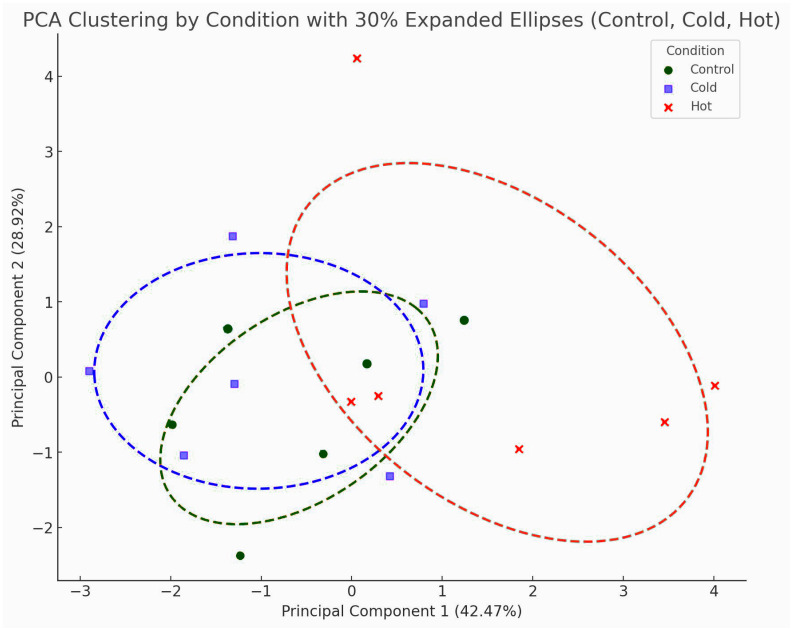
PCA analysis for TPC, MDA, Chl-a, and Chl-b measurements, clustered in three experimental conditions: control, cold, and hot.

The last PCA plot illustrates the clustering of samples under three distinct conditions—control, cold, and hot—with ellipses to encompass the primary spread of data within each group (Figure 5). Together, PC1 and PC2 explain a substantial portion of the dataset’s total variance at 42.47% and 28.92%, respectively. By focusing on these two components, the plot provides a simplified yet informative view of the dominant patterns underlying the variability in the data. Each condition is represented by a distinct marker type (circles for control, rectangles for cold, and crosses for hot), with ellipses indicating the core distribution for each group. Ellipses were drawn around each cluster to indicate the core distribution of samples within that group, encapsulating most of the variability while mitigating the influence of potential outliers. These ellipses help to highlight the degree of overlap or separation among the three conditions. The apparent clustering and separation of the groups suggest that the treatments (in this case, temperature conditions) exert a measurable influence on the underlying variables captured by the PCA. These data indicate that severe heat stress has a major effect on carob physiology.

To gain a deeper understanding of the possible different composition of *C. siliqua* leaves following environmental stress, and as well as to examine the potential antimicrobial activity of the metabolites produced in the leaves of all treatments, both control and stressed leaves were extracted with the same method of using ultrasounds and increasing polarity solvents. Thus, the derived extracts, i.e., DCM (D), MeOH (M), and MeOH-H_2_O:50–50, *v/v* (MW) were first tested for their bioactivity on human pathogenic bacterial and fungal strains and specifically *Bacillus subtilis* DSM10, *Escherichia coli* DSM6897 *Pseudomonas aeruginosa* DSM50071, *Staphylococcus aureus* DSM346, *Candida albicans* DSM1386 and *Saccharomyces cerevisiae* DSM1333, and then their bioactivity against phytopathogenic microorganisms *Xanthomonas campestris* pv. *campestris* 1656 BPIC, *Pseudomonas syrigae* pv. *syringae*, and *Erwinia amylovora* was also examined (see Section 4).

**Figure 6:**
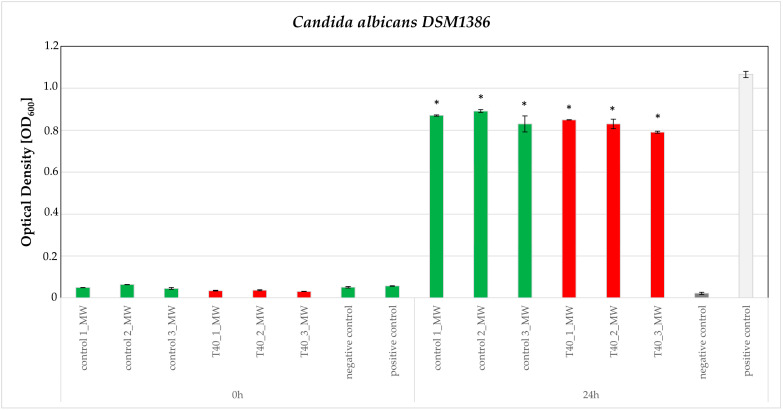
Susceptibility testing of human pathogen *Candida albicans* DSM1386 based on growth in the presence of 200 μg/mL of the control-MW and T40-MW T40 extracts. (Positive control: a culture of the microorganism without added extract; negative control, a culture of the microorganism in the presence of Dimethylsulfoxide—DMSO—10%). The effect is evaluated in OD600 measured at 0 and 24 h after inoculation. Estimated cutoff values and their standard errors for three independent experiments of triplicate datasets are plotted here. Asterisks (*) represent statistically different data points at *p* ≤ 0.05, according to *t*-test comparisons. (Green bars for the control and red for the heat-treated).

**Figure 7:**
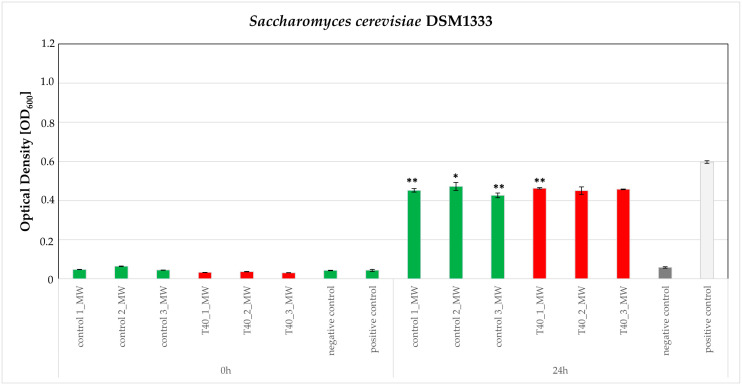
Susceptibility testing of the human opportunistic pathogen *Saccharomyces cerevisiae* DSM1333 based on growth in the presence of 200 μg/mL of control-ΜW and T40-MW extracts. (Positive control: a culture of the microorganism without added extract; negative control, a culture of the microorganism in the presence of DMSO 10%). The effect is evaluated in OD600 measured at 0 and 24 h after inoculation. Estimated cutoff values and their standard errors for three independent experiments of triplicate datasets are plotted here. Asterisks (*) Represent statistically different data points at *p* ≤ 0.05; Asterisks (**) Represent statistically different data points at *p* ≤ 0.01, according to *t*-test comparisons. (Green bars for the control and red for the heat-treated).

According to the susceptibility test of all nine microorganisms, only two human bacterial pathogens and one phytopathogenic bacterium were observed to have growth reduction, caused by the two of the three extracts, i.e., MW and D, respectively. This indicates that polar constituents abundant in MW exhibit an inhibitory effect against human pathogens while non-polar compounds, the components of D extract, are effective against phytopathogens. Furthermore, the stress conditions seem to affect extract potency, signifying qualitative and or/quantitative alternations of their composition. Specifically, control-MW and T40-MW extracts were found to reduce the growth of the human pathogens *C. albicans* DSM1386 and *S. cerevisiae* DSM1333 while T5-MW was found to be inactive. The observed growth reduction was found to be statistically significant compared to the positive control. However, when comparing the growth reduction effects caused by the control and T40-MW extracts, no statistically significant difference was observed (Figure 6 and Figure 7).

**Figure 8:**
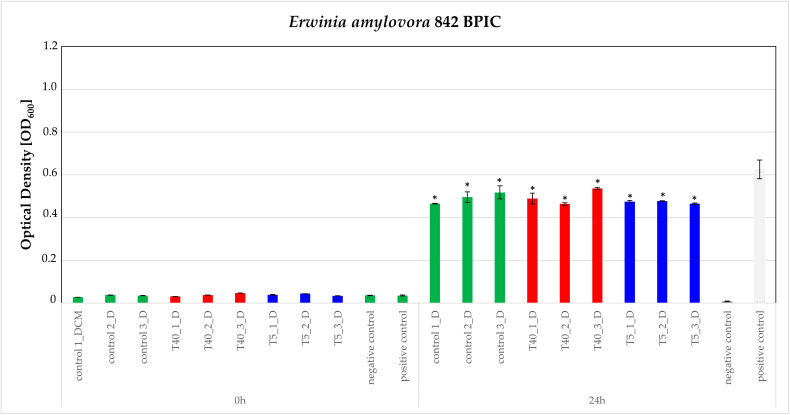
Susceptibility testing of phytopathogen bacterium *Erwinia amylovora* 842 BPIC based on growth in the presence of 200 μg/mL of the control-D, T40-D, and T5-D extracts. (Positive control: a culture of the microorganism without added extract; negative control, a culture of the microorganism in the presence of DMSO 10%). The effect is evaluated in OD600 measured at 0 and 24 h after inoculation using a multi-detection microplate reader. Estimated cutoff values and their standard errors for three independent experiments of triplicate datasets are plotted here. Asterisk (*) represents statistically different data points at *p* ≤ 0.05, according to *t*-test comparisons (Green bars for the control, blue for the cold-treated and red for the heat-treated).

In contrast, control-D, T40-D, and T5-D extracts were found to reduce statistically significantly the growth of the phytopathogen bacterium *Erwinia amylovora* 842 BPIC, compared to the control. The observed growth reduction compared to the positive control (culture of the microorganism without added extract) is statistically significant according to the *t*-test analysis. Moreover, comparing the growth reduction effects caused by the control, T40, and T5 extracts, no statistically significant difference was observed (Figure 8). The other bacterial pathogens were fully grown, with no statistical significance compared to the positive control, when cultured in the presence of the extracts.

The obtained results from the susceptibility test on pathogens oriented the analysis and characterization of the most promising extracts. Thus, MW and D extracts were further investigated. Regarding D extracts, further sample treatment was necessary due to the chemical nature of the components (non-polar). To that end, the D extract was fractionated using LLE to two sub-extracts with the aid of EtOAc and n-Hexane as solvents. The first sub-extract with EtOAc (E extract) is compatible with LC-MS analysis (non-volatile compounds) and the second with n-Hexane (nH) is compatible with GC-MS analysis (volatile compounds). It is important to note that the use of two analytical platforms was necessary to compensate for analytical compatibility aspects originating from the samples’ solubility and polarity [30]. In parallel, the initial M and MW extracts were also analyzed using LC-HRMS/MS to get better insight into the chemical composition of *C. siliqua* leaves.

In specific, suspect analysis was employed to dereplicate known compounds in the different extracts. Moreover, a comparative study between different extracts and stress conditions was undertaken under the metabolite profiling concept aiming to disclose differences and similarities as well as marker compounds associated with stress. In total, 26 compounds were tentatively identified in all samples under investigation (M, MW, and E) using LC-HRMS/MS. The chromatographic and spectrometric data of the identified compounds in *C. siliqua* leaf extracts are presented in Table 3. According to our results, the M and MW extracts were rather similar in composition and mainly consisted of tannins, flavonoid glycosides, benzoic acids, and fatty alcohols, while the E extracts consisted of triterpenoids, methoxyphenols, benzofurans, medium-chain and long-chain fatty acids and glycerophosphoglycerols.

In more detail, the disaccharide maltose (1) and thymine (2) were the first two eluted compounds (0.88 and 0.89 min, respectively). Close in physicochemical properties were the compounds corresponding to peaks 3, 5, 6, 7, 10, 11, and 12, all belonging to the class of metabolites known as tannins (Table 3). These naturally occurring polyphenols can be divided into four primary classes: hydrolyzable tannins (based on ellagic acid or gallic acid), condensed tannins (made of oligomeric or polymeric proanthocyanidins), complex tannins (made of a catechin bound to a gallotannin or elagitannin), and phlorotannins (oligomers of phloroglucinol). Therefore, they were identified as di-galloyl-glucose (three isomers, eluting at 0.94 (3), 1.29 (6), and 1.60 min (7), respectively), glucogallin (5, 1.22 min), tri-galloyl-glucose (10, 4.34 min), and tetra-galloyl-glucose (12, 5.57 min). Peak 11 (5.07 min) was identified as epigallocatechin gallate, a primary metabolite containing a gallate moiety glycosidically linked to a catechin. Epigallocatechin gallate is a typical flavone-3-ol phenolic compound with eight free hydroxyl groups responsible for various biological functions [31].

Furthermore, the flavonoid-3-*O*-glycosides myricitrin (13, 5.73 min) and quercitrin (14, 6.22 min) were detected. HRMS/MS spectra contributed significantly to their identification with the most characteristic pattern the loss of the glucose unit and the detection of the aglycone. Regarding the rest of the chemical classes, they all comprise medium-chain and long-chain fatty acids, fatty amides, fatty acyl glycosides, and linolic acid derivatives. More specifically, after examining the HRMS/MS spectra the detected metabolites were azelaic acid (15, 6.42 min), 5,8,12-Trihydroxy-9-octadecenoic acid (17, 8.39 min), pipericine (18, 8.44 min), LysoPG (19, 16:0/0:0) (8.78 min), 13(S)-Hydroperoxylinolenic acid (20, 8.97 min), octadecenedioic acid (23, 10.34 min). Moreover, gingerol (16, 6.58 min) and two gingerol derivatives were detected, [6]-gingerdiol D-glucopyranoside (21, 9.12 min) and [12]-gingerdiol (24, 11.24 min), as well as two triterpenoids pomolic acid (25, 11.62 min) and ganoderol b (26, 12.32 min).

Regarding the nH sub-extract (fraction of bioactive D extract) and the GC-MS analysis, a total of 21 constituents were tentatively identified, accounting for 76.23% of the total peaks recorded. The stress conditions seem to have a stronger influence on the volatile-nonpolar constituents in comparison to polar ones, as indicated by the GC-MS chromatograms. Among these, glycerolipids played a major role with glycerol 1-palmitate being the most prominent, followed by long-chain fatty alcohols (2-stearoylglycerol). Other minor compounds that were identified were the triterpenoids germanicol, olean-18-ene, beta-amyrone, and beta-amyrine, as well as the lactone (7,9-Di-tert-butyl-1-oxaspiro (4,5) deca-6,9-diene-2,8-dione) and the benzofurans loliolide and dihydroactinidiolide. Additionally, the GC-MS analysis indicated the presence of α-tocospiro A and α-tocospiro B possessing unique 7,8-dimethyl-1-oxaspiro [4.4]-non-7-ene-6,9-dione carbon skeleton.

**Figure 9:**
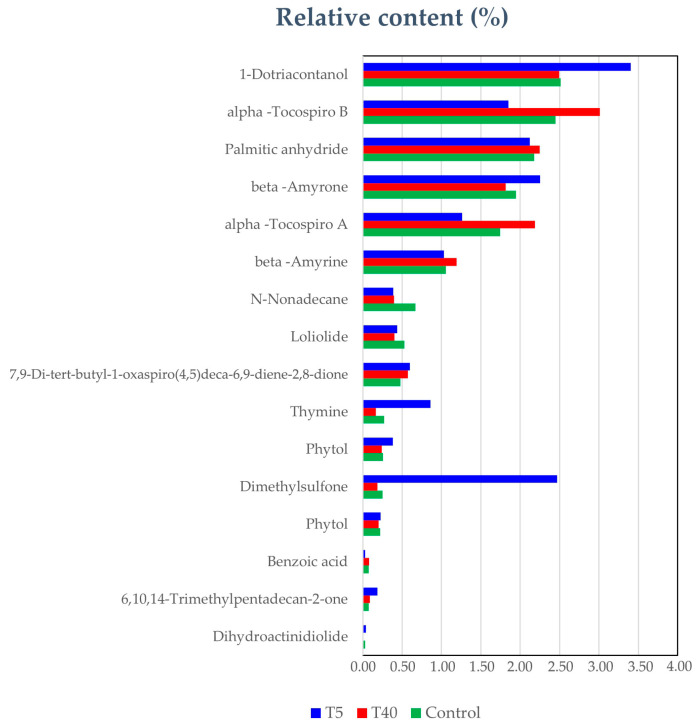
The bar graph of the nH extracts based on the percentage contribution (% peak area) of the metabolites identified between the different stress conditions using GC-MS analysis.

As illustrated in the bar graph of Figure 9, certain compounds seem to be altered by low or high temperatures. Interestingly, the two α-tocopheroids (a-tocospiro A and B), belonging to the general group of tocopherols were found to increase in high temperatures (T40).

**Figure 10:**
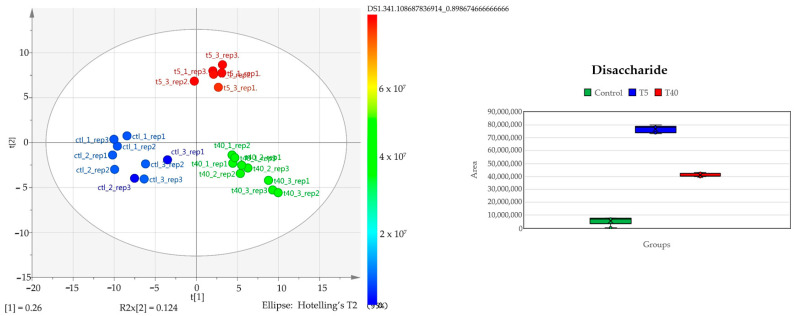
(**Left**): PCA; scatter plot scores of MW extracts. Control leaves samples (blue) and stressed leaves samples [T40 (green) and T5 (red)] are presented; (**Right**): Boxplot indicating the variation of concentration levels of the detected disaccharide 1 amongst the three sample groups.

As mentioned, apart from suspect analysis and dereplication procedure, the LC-MS/MS data were subjected to metabolite profiling to disclose differences between stress conditions and to reveal marker compounds. Multivariate analysis (MVA) was used for descriptive analysis and investigation of trends and classifications based on the secondary metabolites (Figure 10).

**Figure 11:**
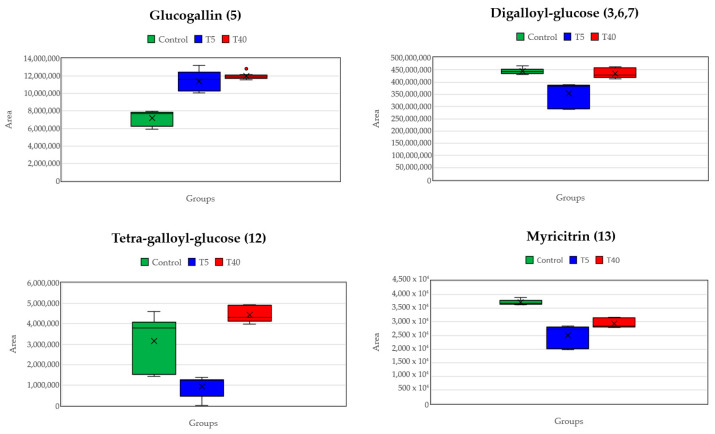
Indicative boxplots of detected compounds presenting statistically significant altered concentration levels under different stress conditions.

It is obvious from the scores plot that the samples are clustered separately, forming three distinct groups. At the first principal component, control and high temperature (T40) are clearly separated, and at the second component, the group of low temperature (T5) is also separated from the other two groups. These trends indicate metabolism alternation under the different treatments. Proceeding with supervised methods and specifically OPLS-DA it was possible to identify statistically significant features which are up- or down-regulated under stress, in comparison with control samples. A notable feature is the elevated levels of a disaccharide, tentatively identified as maltose (1; Table 3), observed in both low- and high-temperature samples compared to the control. From the OPLS-DA plot, it appears that low temperatures have a more pronounced effect. In Figure 11, the metabolites exhibiting the most significant variation due to temperature changes are presented; as illustrated, heat stress conditions alter the hydrolyzable tannin levels. Specifically, high temperatures seem to promote the up-regulation of hydrolyzable tannins, while low temperatures result in reduced levels. Furthermore, the number of esterified gallic acid units on the glucose scaffold might also play a role. It is noteworthy that not all metabolites respond in the same manner when the temperature increases or decreases. Regarding flavonol glucosides, myricitrin, exhibits a reduction at both low and high temperatures, whereas glucogallin, in contrast, increases under cold and hot stress. Furthermore, tetra-galloyl-glucose displays a linear relationship with temperature, meaning that as the temperature rises, its levels also increase, and as the temperature drops, so do its levels. Meanwhile, digalloyl-glucose remains stable at 40 °C but decreases noticeably in colder conditions. These temperature-dependent fluctuations of metabolite concentrations indicate that the plants respond to environmental changes in multiple ways that need further investigation.

## 3. Discussion

Climate change results in the exposure of plants and animals to an abiotic environmental stress to which they are not familiar. Drought, warmer long-lasting summers, and short yet chilling winters, as well as an increase in the global temperature over the years, force all organisms to adapt to the new conditions. Stressing conditions restrict plants’ development and thus their survival fate. In our experiments, carob tree plants chilled for only six days produced less biomass than their control counterparts while the heat-stressed individuals were far more retarded in their final yield. It seems that, although *C. siliqua* is a cold-sensitive species, the high temperatures prove to be far more distressing.

### 3.1. Leaf Structure

It seems that the plants exposed to the chill stress appear less xeromorphic. The plants exposed to heat stress reacted by producing compact leaf tissues with their cells accumulating phenolics; it is known that drought stress results in a decrease in leaf size [32]. The leaves of *C. siliqua* did not seem to exaggerate any of their structural features, compared to some other Mediterranean evergreen sclerophylls [13]. They withstood the high temperatures accumulating phenolics, thus reducing their productivity, while their response to low temperatures was weak and most of the time did not avoid cell injury (Table 1).

The epidermal tissues, which are the thickest among the leaves of the Mediterranean evergreen sclerophylls [13] did not seem to be affected (Figure 2). GC-MS analysis demonstrated the production of α-tocospiro A and α-tocospiro B in heat-stressed samples, both being rare α-tocopherol derivates. It has been well documented that the levels of tocopherols as strong antioxidants, are related to stress tolerance in plants [33] towards the management of excess ROS levels. In contrast, 1-dotriacintanol (fatty alcohols), dimethylsylfone (sulfones), and beta-amyrone (triterpenoids) are up-regulated in cold conditions. All these different chemical classes have been related to the response mechanisms of plants to compensate for abiotic stress [34,35,36]. Tocopherol (Vitamin E) is an antioxidant molecule that is considered to be a major scavenger of lipid peroxyl radicals produced in thylakoid membranes to preserve their structure and function during stress conditions [37,38,39]. Furthermore, tocopherol external application resulted in increased biomass in *Vicia faba* and *Hibiscus rosasineses* due to the enhanced antioxidant capacity of the plants [40,41,42]. In addition, maltose, a disaccharide, occurs in high concentration in cold- and heat-stressed samples when compared to control ones, and is recognized to stabilize the structure of chloroplast stroma in hot (40 °C) and cold stress (5 °C) conditions in *Arabidopsis thaliana* [43]. It has also the ability to protect proteins, membranes, and the photosynthetic electron transport chain at physiologically relevant concentrations under heat stress and freezing stress [43].

Regarding the flavonol glucoside myricitrin (13-Table 3), we monitored this and it was found in lower concentration in exposed samples compared to control in both conditions. While myricitrin is a metabolically or physiologically non-essential metabolite, it may serve as a defense or signaling molecule. This down-regulation relative to the control samples could be associated with metabolic changes triggered by thermal stress. The other flavonol, quercitrin (14-Table 3) breaks down to form quercetin and rhamnose. It is reported that quercetin reduces the level of H_2_O_2_ (required for ABA-induced stomatal closure) that reduces the stomatal closure, which helps the plants face stress in a savior manner [44,45]. Closing of stomata is among the major reactions of Mediterranean plants against summer drought and cold stress [46]. In GC-MS analysis, glycerolipids played a major role with glycerol 1-palmitate being the most prominent, followed by long-chain fatty alcohols (2-stearoylglycerol). It is known that fatty acid production plays a fine-tuning role in drought stress, preserving the structure of cell membranes [47].

### 3.2. Chlorophyll, MDA, and TPC Content

Both chlorophyll-a and -b follow the same concentration pattern. The concentration declines until midday and starts to rise again, to reach the top, in late afternoon. MDA follows a concentration pattern different from that of chlorophyll absorbance (Figure 4). This stress indicator keeps rising during the day to reach the maximum in the late afternoon. This is true for the plants of all three groups. Furthermore, in Figure 3 it is obvious that in the morning, physiology parameters seem to have a more concentrated distribution, indicating the setup of a possible “reset” mechanism, zeroing all environmental affecting factors. The plant, after 6 and 12 h, must face up to environmental pressures leading to oxidative stress and the generation of secondary metabolism to create “safeguard” substances for their survival. Indeed, the plants exposed to high temperatures experience stressful conditions, their response being obvious from late noon to late afternoon. The cold-exposed plants seem to experience stressful conditions as well, yet their response, concerning the accumulation of oxidative factors, seems milder (Figure 5). This augmentation in the MDA concentration following heat stress is in accordance with ref. [48], where heat-stressed (32 °C) seedlings of *C. siliqua*, suffering drought stress, reported an increase in MDA content. Moreover, signs of lipid peroxidation damage were detected in heat-treated plants, in which drought caused an increase of 40% in malondialdehyde (MDA) content. Epigallocatechin gallate, a phenolic compound responsible for various biological functions [31], is synthesized via the flavonoid biosynthesis pathway. It is one of the most important secondary metabolites that can relieve plants from temperature stress. In tea plants, a 6-day exposure to 35 °C increased the concentration of epigallocatechin gallate, pointing out its role in the adaptation to high temperatures [49]. Exogenous induced epigallocatechin gallate relieved tea plants from heat stress, leading to augmentation of apoplastic ROS signaling; the same motif is true for tomato plants; exogenous epigallocatechin gallate resulted in apoplastic ROS production, offering a resistance to tobacco mosaic virus (TMV) [50]. Furthermore, epigallocatechin gallate also emerges as a safeguard metabolite for cold stress, increasing ROS and MDA levels in tea plants [51]. According to Keunen et al. [52] there is a strong relationship between sugars and especially disaccharides with stress-induced ROS accumulation in plants. In our data, epigallocatechin gallate seems to remain in the same concentration in both treatments; this fact indicates that *C. siliqua* can withstand stress.

The TPC seems to follow the pattern of the chlorophyll-a and -b content in the plants of all three groups. Considerable differences in the diurnal concentration were observed in the plants of the heat-stressed group. This diurnal variation of phenolics means that not all of them are terminal metabolic products, i.e., tannins, but some of the phenolics are hydrolyzable and present a degree of mobility, probably of high importance for the plant. Phenolic compounds are classified into flavonoids and non-flavonoids [53]. Phenolic acids (e.g., gallic acid) function as precursor molecules of hydrolyzable tannins. Tannins and phenolic acid are non-flavonoids. Tannins are amongst the most characteristic classes of polyphenols that have been reported for their implication in plant stress tolerance [54]. Figure 5 PCA plot provides a clear visual of how each condition influences the overall data structure, suggesting condition-specific patterns. Heat stress seems to provoke a greater effect on plants’ physiology, indicating that extreme prolonged temperature affects oxidative stress and the accumulation of phenolics. Heat stress conditions in *Festuca trachyphylla* favor the increased expression of hydrolyzable tannins and phenolic acids [55,56,57]. The accumulation of tannins is considered to be the factor that enhances plants’ resistance after ROS scavenging [58]. This is in accordance with our data, in which MDA concentration and TPC appeared not to follow the same pattern but were time-delayed (Figure 4).

Given that the tissues of *C. siliqua* can survive in a narrow span of low temperatures, from above −6 °C for the leaves to above −11 °C for the stem xylem, compared to the other Mediterranean evergreen sclerophylls (Table 3) [59], we may assume that the plant has not invested in overcoming the chilling effects thus the response of the cold-stressed plants is rather inconspicuous.

### 3.3. Bioassays

It is important to note that MW extracts from the control and heat-stressed samples could inhibit the growth of *C. albicans* and *S. cerevisiae*, human pathogen strains. On the other hand, inhibition of the growth of phytopathogen Gram-negative bacterium *Erwinia amylovora,* following incubation with D extracts (control, T40, and T5) was also notable. The consequent incubation with the two sub-extracts of D (nH and E), did not exhibit any significant effect on the inhibition of *Erwinia amylovora* growth. That was not expected and could imply that the observed activity is the result of a synergistic effect commonly reported for complex mixtures such as plant extracts. Further deconvolution of this complexity is required to reveal the active compound and/or compounds [57,58], and *E. amylovora* is considered the “guilty” one for the fire blight disease on many individuals of the Rosaceae family [60]. Therefore, it is important that D extract from *C. siliqua* leaves, control or heat- and cold-stressed, could inhibit the growth of this bacterium and could have a drastic effect on relieving the plants that suffer from the “attack” of *E. amylovora*.

## 4. Materials and Methods

### 4.1. Plant Material and Exposure Setup

**Figure 12:**
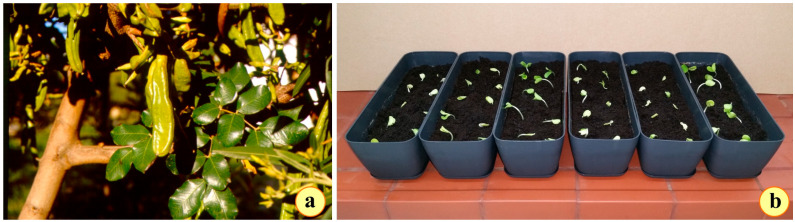
(**a**) The leaves and the pods containing the seeds of a wild growing tree of *Ceratonia siliqua*; (**b**) The pots with the carob tree plantlets at the beginning of the experiment.

The seeds of *Ceratonia siliqua* are brown, with extremely tough, impermeable seed coats exhibiting external physical dormancy impeding water absorption [61], thus hindering germination [62,63]. They sprout, in nature, either from an entire pod buried in the soil or from seeds that had been consumed by certain bird species, digested, and eliminated far away. They also sprout, eventually, after wildfires, which are very frequent in the areas where this species occurs. In the laboratory, they do not sprout unless they undergo a dormancy-breaking treatment. Pods of *C. siliqua* (Figure 12a) were collected from the island of Ios, Greece (36°44′02.8″ N 25°16′37.1″ E). They were crushed manually to extract the seeds. For the current experiment, acid scarification was used. Seeds of *C. siliqua* were immersed in 65% sulphuric acid for 10 min [64,65]. The treated seeds were washed for 30 min with running tap water, dried, and drenched with fungicidal powder [Thiram; (Tetramethylthiuram disulfide)–IUPAC name: dimethylcarbamothioylsulfanyl N,N-dimethylcarbamodithioate—U244 EPA code, Sigma-Aldrich, St. Louis, MO, USA]. One hundred seeds were imbibed in three Petri dishes, each one containing two sheets of filtered paper, and grown at 22°C (70% humidity), in a light/dark cycle of 16 h/8 h and 110 μmol/m^2^/s of (PAR) illumination supplied by cool-white, fluorescent tungsten tubes (Osram, Germany). The germination procedure was completed in 5 days, scoring almost 99% (98 seeds out of 100 were germinated; 1 was empty). Germinated seeds (radicle protrusion about 1 cm) were sown in 30 × 10 × 10 cm, 3.0 L PVC long pots trays filled with wet Potgrond P medium (Klasmann–Deilmann), at pH 6.0 (Figure 12b), with 10 seedlings in each pot tray. Initially, two pot trays with 10 seedlings each were placed to grow in each one of the three culture chambers. To ensure the possibility of replacing any loss during the 6-week adaptation period and prior to the application of the stress conditions, an additional pot tray with 10 seedlings as well was added to the “control” culture chamber; however, after all, there was no need for using any additional seedlings. Two pot trays (tray 1 and tray 2) were incubated in each one of the temperature-controlled plant growth culture chambers (elvem–model BOD100, Spata 19004, Athens, Greece).

**Figure 13:**
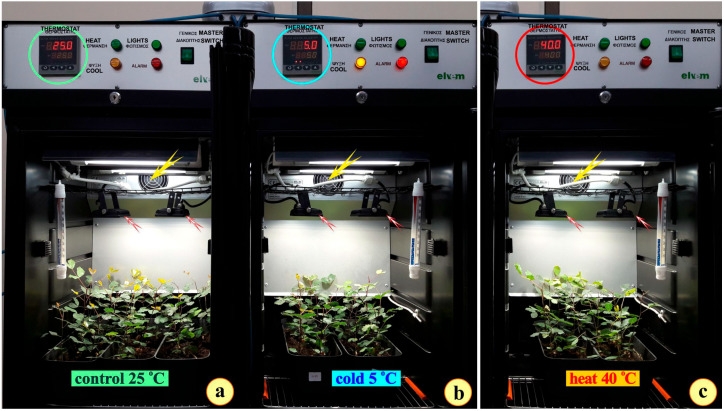
The experimental setup and the plants of *Ceratonia siliqua* at the end of the experiment; (**a**) the plant growth culture chamber with the two pot trays of the control plants and the additional spare pot tray. The temperature was adjusted to 25.0 °C (T25 plants) (green cycle); (**b**) The culture chamber with the cold-stressed plants. The temperature adjusted to 5.0 °C (T5 plants) (cyan cycle); (**c**) The culture chamber with the heat-stressed plants. The temperature adjusted to 40.0 °C (T40 plants) (red cycle). The small white arrows point at the 10 W, 1000 lumen LED floodlights; the yellow arrows point at the temperature-stabilizing fan.

The culture chambers have built-in illumination sources: two 1000 lumen, 10 W LED at 4500 K assisted by two Osram basic, T5 Short, L 6 W/640 miniature tubes, length 212 mm, diameter 16 mm, 4000 K. Total illuminance at the surface of the pots, directly measured with a MASTECH MS6610 portable luxmeter (MASTECH Group, Taipei, Taiwan), was 125.7 to 128 μmol·m^−2^·s^−1^ (Figure 13a–c).

Air was supplied through a HAILEA ACO-9160 (Chaozhou, China) air pump, at an output of 2 L/min for each incubator. All incubators were initially set at 25.0 °C (±0.2), a temperature reported to be appropriate for the germination and growth of *C. siliqua* [55,59]. The incubator temperature was constantly monitored with an industrial, two-channel data logging (Model AZ9882, AZ Instrument Crop., Taichung, Taiwan) thermometer (probe sensitivity: approximately 43 µV/°C) through the attached Type T (copper–constantan) needle-shaped thermocouples.

To ensure that all plants in each incubator received the same light energy, we changed the position of each pot tray at 09:00 every morning, so that the pot trays alternated from being, each, either at the left, in the middle, or at the right side within the incubator. The seedlings of all three groups were left to grow for six weeks at a 24 h cycle of 25 °C (adaptation period). Then, the 6-day temperature stress period followed. The temperature in each incubator was adjusted as follows: 25.0 °C (24 h cycle) for the control plants (T25) (Figure 13a); 5.0 °C (24 h cycle) for the cold-stressed plants (T5) (Figure 13b) and 40.0 °C (24 h cycle) for the heat-stressed plants (T40) (Figure 13c). The pots in each of the three chambers received 110 μmol/m^2^/s of (PAR) illumination for a light/dark cycle of 16 h/8 h, for six days and nights. At the end of this period, sampling for chlorophylls, total phenolic content, and MDA estimation were performed as follows: (1) by the end of the experiment (08:00), (2) at 14:00, (3) at 20:00, and for the next day (4) at (08:00), (5) at 14:00, and (6) at 20:00.

### 4.2. Microscopy

At the end of the experiment, the pot trays were removed from the three culture chambers and their contents were dispersed in water to remove the culturing substrate from the roots of the plants.

Small parts, adjacent to the central nerve, were removed at random from the middle of the younger, upper pair leaves on each plant, cut into small pieces (1 × 1 mm), and fixed in phosphate-buffered 3% glutaraldehyde (pH 6.8) at 0 °C for 2 h. The tissue was post-fixed in 1% osmium tetroxide in phosphate buffer and dehydrated in graded ethanol series. The properly prepared pieces were either (a) transferred in 100% acetone, critical point dried, coated with gold, and viewed with a JEOL JSM-6360 Scanning Electron Microscope (JEOL Ltd., Tokyo, Japan), or (b) embedded in Durcupan ACM (Fluka, Steinheim, Switzerland).

Stomatal frequency was measured on the peeled abaxial epidermis of 5 mature leaves, in 30 different optical fields for each leaf. Average stomatal frequency as well as standard deviation were calculated. Statistical analysis using the Tukey test was performed for the average stomatal frequency.

Semithin sections (5–6 microns) obtained from an LKB Ultrotome III (Stockholm-Bromma 1, Sweden), were placed on glass slides, and stained with 0.5 toluidine blue O (in 1% borax solution), as a general stain, for light microscopic observations. Ultrathin sections (50–60 nm) were placed on 100 mesh grids, double stained with uranyl acetate and lead citrate [66], and viewed with a Phillips EM-300 Transmission Electron Microscope (Phillips, Amsterdam, The Netherlands). The literature on double fixation is cited in detail [67,68].

### 4.3. Pigments Protocol

Total chlorophyll-a and -b (Chl-a and Chl-b) content was spectrophotometrically determined. Approximately 50 mg fresh leaves, 10 leaf samples from each treatment, were extracted with 1 mL 80% (*v/v*) acetone, overnight, at 4 °C. Supernatant was transferred to a 1,5-mL glass cuvette for measurement in v-1200 spectrophotometer (WVR; Pennsylvania, PA, USA). Absorbance was read at both 663.6 and 646.6 nm, corresponding to chlorophyll- and chlorophyll-b respectively. Quantification of pigment content was calculated using molar extinction coefficients specifically for this method and normalized per fresh weight (mg/g) [69]. Chlorophyll-a, e_663.6_ = 76.79 and e_646.6_ = 18.58; chlorophyll–b, e_663.6_ =9.79 and e_646.6_ = 47.04 [70]. All data was expressed as the mean of ten samples ± standard error of the mean.

### 4.4. Protocol for MDA (Malondialdehyde) Determination in Plant Tissues

Extraction of MDA from 50 mg plant material (frozen and ground in liquid nitrogen) was executed using 1 mL 0.25% thiobarbituric acid (TBA—Sigma-Aldrich™, Milan, Italy) dissolved in 10% trichloroacetic acid (TCA—Sigma-Aldrich™, Milan, Italy). The extract was collected in a 1.5-mL Eppendorf tube, and the mixture was heated at 85 °C for 30 min, and then quickly chilled on ice. The same procedure was followed for the blank sample but without plant material. The mixture was centrifuged at maximum speed for 10 min to pellet the particles. The supernatant was then transferred in a 1-mL plastic cuvette for spectrophotometric measurement. The absorbance was determined primarily at 532 nm (the peak of the MDA–TBA complex) and then at 600 nm (nonspecific absorption). 1 mL 0.25% TBA in 10% TCA was used as a blank. Calculate *A*_(532–600)_ [71]. The MDA concentration was estimated using the Beer–Lambert–Bouguer law, MDA extinction coefficient ε_532_ 155 mM^−1^ cm^−1^; the amount of MDA was calculated; and the values were normalized to the fresh weight of each sample.

### 4.5. Determination of Total Phenolic Content

Total phenolic content (TPC) in carob leaf extracts was determined using the Folin–Ciocalteu colorimetric method [72,73]. Dry tissue (0.1 g) from 10 leaf samples from each treatment was ground in an electric mill (Janke & Kunkel-Mikro-Feinmuhle-Cullati, IKA Labortechnik, Wasserburg, Germany) and immersed in 10 mL MeOH (50% *v/v*). The tissue was incubated in a water bath for 3 h at 40 °C and vortexed regularly. Phenolic compounds were extracted, filtered using Whatman ^®^ (Maidstone, UK) #2 filter paper), and kept tightly sealed at 4 °C overnight. Furthermore, an aliquot (0.05 mL) of the diluted leaf extract [1:5 MeOH 10% (*v/v*)] was added to a solution consisting of 3.95 mL of dH_2_O, 0.25 mL Folin–Ciocalteu (Sigma-Aldrich™, Milan, Italy) reagent (that was previously diluted with water 1:10 *v/v*), 0.75 mL Na_2_CO_3_ 20% (*w/v*) and was vortexed for 30 s. The solution was kept at 20 °C for 2 h and the absorption of the resulting colorimetric reaction was measured with a UV–VIS spectrophotometer (Pharmacia Biotech Novaspec II, Uppsala, Sweden) at 760 nm. Calculation of the total phenolic content was performed using standard curves of tannic acid and expressed as mg of tannic acid equivalent per g (dry weight) of leaf tissues. To create the standard curve, the procedure was as follows; 0.05 mL of the different tannic acid concentrations, that is, 0.02 mg/mL, 0.08 mg/mL, 0.16 mg/mL, and 0.40 mg/mL, were added to 3.95 mL of dH_2_O, 0.25 mL Folin–Ciocalteu reagent (previously diluted with water 1:10 *v/v*), 0.75 mL Na_2_CO_3_ 20% (*w/v*), and vortexed. The absorbance of these solutions, following the colorimetric reaction (after 2 h at 20 °C), was measured with a UV–VIS spectrophotometer (Pharmacia Biotech Novaspec II, Uppsala, Sweden) at 760 nm; the absorbance was plotted versus the concentration for the standard curve.

### 4.6. Mass Spectroscopy (HRMS) Analysis

Leaves of *C. siliqua* L. were dried at room temperature and ground to a fine powder using a laboratory blender to achieve sample uniformity and protect the initial raw material from infections and chemical degradations. Then, extractions were carried out via ultrasound-assisted extraction (UAE). In brief, 3 g of each sample were mixed in a ratio of 1:10 with solvent and kept in the ultrasound bath for 20 min, at 30 °C. Three successive extractions were performed for each sample. Initially, samples were extracted with dichloromethane (DCM), then methanol (MeOH), and finally with water (H_2_O). Samples were filtered and evaporated until dryness. Extractions were performed in triplicate for each sample, while aliquots were used for the subsequent analyses. Furthermore, the DCM fraction was subjected to an additional liquid–liquid extraction step with ethyl acetate and n-hexane (EtOAc/n-Hex 1:1 *v/v*). The EtOAc extracts were concentrated until dry in a rotary evaporator under reduced pressure at 30 °C followed by their analysis with UPLC-ESI (±) Orbitrap-MS. Additionally, aliquots of the n-hex fraction were used for the subsequent GC-MS analyses.

UPLC-HRMS and HRMS/MS analysis was conducted on a Waters Acquity^®^ UPLC system (Waters Corporation, Milford, MA, USA) coupled with an LTQ-OrbitrapR XL hybrid mass spectrometer (Thermo Scientific, Waltham, MA, USA). To optimize UPLC conditions, eluants, column temperature, and column properties were examined. Improved peak shape was achieved with a combination of acetonitrile (ACN), acidified water, and a column temperature of 40 °C. The mass spectrometer, equipped with an electrospray ionization (ESI) source, operated in both negative and positive modes. The extracts were analyzed with an LC gradient consisting of H_2_O with 0.1% formic acid (FA) (solvent A) and ACN (solvent B). The samples were dissolved in the mobile phase (90% B and 10% A). The gradient elution was conducted with 95% (A) and 5% (B) as initial composition and was maintained for 1 min. For the next 15 min, the organic phase (B) was increased to 100% and this composition remained for 2 min before the initial conditions were restored for equilibration purposes. The total running time was 20 min. Throughout the run, the flow rate was maintained at 400 μL min^−1^ and the injection volume was set to 10 μL.

High-resolution mass spectra were acquired in the full scan *m/z* range of 113–1000, in negative and positive mode at a resolving power of 30,000 and data-dependent MS/MS events were acquired, using an electrospray ionization source (ESI). In both modes, the data-dependent acquisition involved collision-induced dissociation C-trap (CID) with a normalized collision energy set at 35 V and a mass resolution of 10,000. In negative mode capillary temperature was adjusted to 350 °C and the source voltage was 2.7 kV, with tube lens and capillary voltage tuned at −100 V and −30 V, respectively. In positive mode capillary temperature was set to 350 °C and the source voltage was 3.60 kV, with tube lens and capillary voltage tuned at +120 V and +40 V, respectively. System control and spectral interpretation were carried out using the XcaliburTM (Version 2.2, Thermo Scientific) software. For identification and dereplication, the following ranking criteria were applied: Rt, EC, 10 ≤ RDBeq ≤ 20, mass range *m/z* 100–1000 Da, mass tolerance ≤ 5 ppm; HRMS/MS spectra of the selected peaks and identification of the fragmentation pattern, as well as literature references were consulted for further confirmation.

### 4.7. Data Processing and Chemometrics

After LC-HRMS/MS acquisition, the data was processed in order to be subjected to multivariate data analysis (MVA) for the generation of descriptive statistical models. HRMS/MS data was recorded with Xcalibur 2.2. and raw files (.raw, Thermo) were imported to MZmine 2.53 software for data processing. The peak list was generated with a centroid selection algorithm. For chromatogram building of the generated mass list, 0.05 min was set as the minimum time of span and 5 ppm for mass tolerance. A chromatogram deconvolution module was employed, and spectra were processed with a local minimum search algorithm. The minimum retention time range was set at 0.1 min and the peak width at 0.05–0.7 min. Chromatograms were aligned, and spectra were normalized regarding retention time with 0.005 min tolerance. The joint aligner algorithm was applied to detected masses using a match score, calculated based on the mass and detection time of each peak, was used. Finally, gap filling was implemented, using the peak finder method.

Both peak lists (positive and negative mode) were imported to SIMCA 14.1 (Umetrics, Umeå, Sweden) software for statistical analysis. Mainly, Principal Component Analysis (PCA) and Orthogonal Partial Least Squares-Discriminant Analysis (OPLS-DA) were implemented for sample visualization and discrimination, while s-plots were built between groups for determination and identification of statistically significant metabolites responsible for the observed trends and classifications. For this purpose, *p*-values in PCA and Variable Importance in Projection (VIP) values of OPLS- DA models, which rank variable contribution, were estimated, and evaluated. *p* < 0.05 and VIP scores > 1 were considered statistically significant. The generated models were evaluated for their R2 and Q2 parameters indicating the goodness of fit and predictability, respectively. Only models with R2 values close to 1, Q2 values over 0.5, or models with lower R2 but close to Q2 value were accepted. A permutation test was also applied for further validation of the models. Similarly, only models that succeeded in the permutation test were used for data visualization and subsequent VIP calculations.

### 4.8. Gas Chromatography–Mass Spectrometry (GC-MS) Analysis

Carob leaves n-Hex extracts underwent chemical analysis utilizing gas chromatography–mass spectrometry (GC-MS) using an Agilent 8890 GC system (Agilent Technologies, Santa Clara, CA, USA), equipped with an Agilent 7693A autosampler (Agilent, Santa Clara, CA, USA), an HP-5MS capillary column (30 m, 0.25 mm i.d., 0.25 μm film thickness) and an Agilent 5977B GC/MSD mass spectrometer. Helium served as the carrier gas with a flow rate of 1 mL/min. Column temperature initiated at 60 °C for 5 min, then gradually increased to 280 °C at a rate of 3 °C/min, maintaining that temperature for an additional 10 min. Electron ionization (EI) with an ionization energy of 70 eV was employed for MS detection. Prior to injection to the GC-MS, 0.4 mg of each extract was diluted in 1 mL of n-hexane and sent for analysis. The injection volume was 1.0 μL and diluted samples were injected automatically in splitless mode. Freshly prepared samples in triplicate were analyzed just before the analysis. Alkane C8–C20 in hexane served as a standard marker. Data was acquired and processed using MassHunter Workstation Qualitative Analysis Software (version 10.0). Compound identification involved comparing obtained mass spectra with available spectral libraries (Adams07, Nist20, and Wiley275), as well as through comparison of their relative retention indices (RRIs) with ones found in the literature.

### 4.9. Strains of Microorganisms and Growth Conditions

Bacterial strains included *Bacillus subtilis* DSM10, *Escherichia coli* DSM6897, *Pseudomonas aeruginosa* DSM50071, *Candida albicans* DSM1386, *Staphylococcus aureus* DSM346, *Saccharomyces cerevisiae* DSM1333, and *Xanthomonas campestris* pv. *campestris* 1656 BPIC, *Pseudomonas syringae* pv. *syringae*, *Erwinia amylovora* 842 BPIC (*X. campestris* pv. *campestris* 1656 BPIC, *P. syrigae* pv. *syringae*, *E. amylovora* 842 BPIC were obtained from the Benaki Phytopathological Institute Collection (BPIC) (https://cc.info.wdcm.org) (Table 4).

### 4.10. Broth Microdilution Method

The broth microdilution technique was used to determine the antimicrobial potential of the extracts against the nine indicator strains, as given in Table 4. The method was followed as described [74] with the following alterations: DMSO 10% (*w/w*) was used as reference bactericide and the cytotoxicity of the solvents was also tested, at the maximum concentration they had in the final solutions. All plates were incubated for 24 h and OD absorbance (600 nm) was measured at 0 and 24 h after inoculation in triplicate with 10 s shaking UV/VIS on a monochromatic microplate spectrophotometer (Multiskan GO, Thermo Fisher Scientific Corporation: Waltham, MA, USA). The effect of the extracts on the growth of the indicator strains was evaluated by the reduction or not of the growth of the indicator strains, in the presence of 100 μg/mL, 200 μg/mL, and 500 μg/mL of the extracts.

### 4.11. PCA Analysis

For the statistical analysis of chlorophyll-a and -b, MDA, and TPC. All data was expressed as the mean of 10 samples ± standard error of the mean. From each treatment, 20 leaves (2 leaves per seedling) were used per replication; 20 leaves from pot tray 1 and 20 leaves from pot tray 2. All data were expressed as the mean of 2 replicates ± standard error of the mean (*N* = 20, *n* = 2). Data were previously checked for their normality, while the Tukey test was evaluated using OriginPro v.9.1 and MS Excel, for the statistical significance. Then data was subjected to Principal Component Analysis (PCA) using Python and the scikit-learn library [75] to reduce data dimensionality and identify primary variance components.

## 5. Conclusions

Carob is one of the most common trees of the Mediterranean basin and has been a part of the culture and eating habits for thousands of years. Our data demonstrate that no “impressive” changes were observed in the morphology, anatomy, and physiology of cold- or heat-stressed samples. Furthermore, based on the metabolite profiling approach and MVA, it seems that there is an impact on metabolome under stress conditions, and certain marker compounds were revealed as belonging to tannins and flavonoids. Moreover, non-polar/volatile compounds belonging to α-tocopheroids, fatty alcohols, sulfones, and triterpenoids were found to be altered either in high or low temperatures. However, the carob tree presents only a small shift of its metabolism to anticipate stressing conditions. Furthermore, the MeOH-H_2_O (MW-polar constituents) leaf extracts presented notable activity against the human pathogens *Candida albicans* and *Saccharomyces cerevisiae*, while DCM (D-non-polar constituents) extracts inhibited the growth of the phytopathogenic *Erwinia amylovora*. This data, along with recent [76], demonstrated that carob trees can grow in different polluted environments, indicating that *C. siliqua* is the most adapted Mediterranean plant, with “sophisticated” reactions. This aspect could upgrade the concept of the carob tree as a useful plant for green city creation along with its use in poor/drought soils and arid/semi-arid environments currently, as climate change is becoming a crisis.

## Figures and Tables

**Table 1:** Frost resistance of vegetative organs of adult specimens of Mediterranean sclerophylls and conifers in December and January. Data indicate temperatures (°C) that cause 50% injury. Adapted from [8,9].

Species	Leaves	Leaf Buds	Stem Cambium	Stem Xylem
*Ceratonia siliqua*	−6	−8	−9	−11
*Nerium oleander*	−8	−12	−14	−15
*Myrtus communis*	−12	−11	−17	−15
*Laurus nobilis*	−12	−10	−14	−16
*Olea europaea*	−12	−13	−20	−18
*Quercus coccifera*	−12	−13	−21	−22
*Quercus suber*	−12	−16	−26	−22
*Arbutus unedo*	−12	−17	−18	−16
*Rhamnus alaternus*	−12	−18	−17	−16
*Viburnum tinus*	−13	−15	−20	−17
*Pistacia lentiscus*	−14	−16	−20	−17
*Quercus ilex*	−15	−17	−28	−26
*Phillyrea latifolia*	−16	−20	−23	−22
*Pinus pinea*	−13	−16	−19	−17
*Pinus halepensis*	−13	-	−22	−18
*Cupressus sempervirens*	−16	-	−29	−22

**Table 2:** Data on the thickness of the leaf tissues. (The results with a statistically significant difference are grouped using the compact letter display (CLD) methodology; *p* ≤ 0.05, according to Tukey test comparisons).

	μm	Thickness of the Adaxial Epidermis	Thickness of the Abaxial Epidermis	Thickness of the Palisade Parenchyma	Total Leaf Thickness
Treatment					
*C. siliqua* control		43 ± 7 ^a^	27 ± 6	84 ± 12	282 ± 15 ^a^
*C. siliqua* cold-treated		30 ± 5 ^b^	20 ± 5	69 ± 15	180 ± 12 ^b^
*C. siliqua* heat-treated		38 ± 3 ^ab^	24 ± 6	75 ± 11	213 ± 16 ^b^

**Table 3:** UHPLC-Orbitrap-MS data of *C. siliqua* L. leaves extracts identified compounds. RT: Retention time expressed in minutes; exp. m/z: Experimental m/z of pseudomolecular ion; Theoretical m/z; Δm: mass accuracy expressed in ppm. RDBeq.: Ring and double bond equivalent; Spectrometric information for characteristic HRMS/MS fragment ions are also given.

ID	Mode	Compound	RT (min)	Exp. *m/z*	Theor. *m/z*	Δm (ppm)	Formula	RDB	Fragment Mass	Chemical Class
1	Neg	Disaccharide (Maltose)	0.88	341.10904	341.1089	0.4	C_12_H_22_O_11_	2.5	179.0562/161.0456/281.0885	Carbohydrates and carbohydrate conjugates
2	Pos	Thymine	0.89	127.0502	127.0502	0.0	C_5_H_6_N_2_O_2_	3.5	84.0445/109.0399	Pyrimidines and pyrimidine derivatives
3	Neg	**Di-galloyl-glucose (isomer 1)**	0.94	483.0771	483.0769	0.4	C_20_H_19_ O_14_	11.5	331.0667/313.0562/169.0140	Tannins
4	Pos	Loliolide	1.13	197.1172	197.1172	0.0	C_11_H_16_O_3_	3.5	179.1068/135.1169/161.0962	Benzofurans
5	Neg	**Glucogallin**	1.22	331.0664	331.066	1.2	C_13_H_15_O_10_	6.5	271.0456/211.0245/169.0140	Tannins
6	Neg	**Di-galloyl-glucose (isomer 2)**	1.29	483.0771	483.0769	0.4	C_20_H_19_O_14_	11.5	331.0667/313.0562/169.0140	Tannins
7	Neg	**Di-galloyl-glucose (isomer 3)**	1.60	483.0771	483.0769	0.4	C_20_H_19_O_14_	11.5	331.0667/313.0562/169.0140	Tannins
8	Neg	6-O-(hydroxybenzoyl)hexopyranosyl 6-O-(trihydroxybenzoyl)hexopyranoside	2.5	443.1191	443.1184	1.6	C_19_H_23_O_12_	8.5	331.0669/313.0564/193.0141	
9	Neg	Methyl gallate	4.23	183.0296	183.0288	4.4	C_8_H_7_O_5_	5.5	x	Benzoic acids and derivatives
10	Neg	Tri-galloyl-glucose	4.35/5.04	635.0877	635.0879	−0.3	C_27_H_23_O_18_	16.5	465.0668/421.0773	Tannins
11	Neg	epigallocatechin gallate	5.07	457.07742	457.0765	2.0	C_22_H_17_O_11_	14.5	x	Flavans (catechin gallates)
12	Neg	**Tetra-galloyl-glucose**	5.57	787.0984	787.0988	−0.5	C_34_H_27_O_22_	21.5	635.0887/617.0782/465.0676	Tannins
13	Neg	**Myricitrin**	5.73	463.0877	463.0871	1.3	C_21_H_19_O_12_	12.5	317.0298/316.0221	Flavonoid glycosides
14	Neg	Quercitrin	6.22	447.0928	447.0922	1.3	C_21_H_19_O_11_	12.5	301.0352	Flavonoid glycosides
15	Neg	Azelaic acid	6.42	187.0976	187.0976	0.2	C_9_H_16_O_4_	2.5	125.0971/143.1077/169.0871	Medium-chain fatty acids
16	Pos	Gingerol	6.58/10.09	293.1758	293.1758	0.0	C_17_H_26_O_4_	5.5	221.1547	Methoxyphenols
17	Neg	5,8,12-Trihydroxy-9-octadecenoic acid	8.39	329.2333	329.2333	−0.1	C_18_H_34_O_5_	2.5	311.2231/171.1027	Long-chain fatty acids
18	Pos	Pipericine	8.44	336.3260	336.3261	−0.3	C_22_H_41_NO	2.5	319.3001/301.2896	Fatty amides
19	Pos	LysoPG(16:0/0:0)	8.78	485.2885	485.2874	2.3	C_22_H_45_O_9_P	0.5	351.1803/467.2787	Glycerophosphoglycerols
20	Neg	13(S)-Hydroperoxylinolenic acid	8.97	309.2071	309.2071	0.0	C_18_H_30_O_4_	4.5	291.1967/239.1655/183.1029/273.1864	Lineolic acids and derivatives
21	Neg	[6]-Gingerdiol D-glucopyranoside	9.12	457.2442	457.2443	−0.2	C_23_H_38_O_9_	5.5	181.0719	Fatty acyl glycosides
22	Pos	MG(16:0/0:0/0:0)	10.18	313.2734	313.2743	−2.9	C_19_H_38_O_4_	1.5	257.2477/239.2371	Monoradylglycerols
23	Neg	Octadecenedioic acid	10.34	311.2227	311.2228	−0.3	C_18_H_32_O_4_	3.5	293.2124	Long-chain fatty acids
24	Pos	[12]-Gingerdiol	11.24	381.2995	381.2999	−1.0	C_23_H_40_O_4_	3.5	265.2556/165.1638/135.1169/121.1012	Methoxyphenols
25	Neg	Pomolic acid	11.62	471.3483	471.3480	0.6	C_30_H_48_O_4_	7.5	425.3427/339.2694	Triterpenoids
26	Pos	Ganoderol B	12.32	423.3613	423.3627	−3.3	C_30_H_48_O_2_	7.5	405.3515/189.1638/283.2421	Triterpenoids

**Table 4:** Indicator strains, growth, and culture conditions.

Indicator Strains	Media	Incubation Temperature	Accession Number
*Bacillus subtilis*	Luria-Bertani (LB) agar/broth	30 °C	DSM10
*Escherichia coli*	Luria-Bertani (LB) agar/broth	37 °C	DSM6897
*Pseudomonas aeruginosa*	Luria-Bertani (LB) agar/broth	30 °C	DSM50071
*Candida albicans*	YPD agar/broth	37 °C	DSM1386
*Staphylococcus aureus*	Luria-Bertani (LB) agar/broth	37 °C	DSM346
*Saccharomyces cerevisiae*	YPD agar/broth	30 °C	DSM1333
*Xanthomonas campestris* pv. *campestris*	Luria-Bertani (LB) agar/broth	30 °C	1656 BPIC
*Pseudomonas syrigae* pv. *syringae*	Luria-Bertani (LB) agar/broth	30 °C	-
*Erwinia amylovora*	Luria-Bertani (LB) agar/broth	30 °C	842 BPIC

## Data Availability

The original contributions presented in the study are included in the article, further inquiries can be directed to the corresponding author.
